# Nasal reconstruction: Nasal Alar Rim Notching Deformity Reconstruction With Auricular Composite Chondrocutaneous Graft

**Published:** 2018-12-20

**Authors:** Spencer M. Jackson, Tom Reisler

**Affiliations:** ^a^Department of Surgery, The Brody School of Medicine, East Carolina University, Greenville, NC; ^b^Division of Plastic and Reconstructive Surgery, Department of Surgery, The Brody School of Medicine, East Carolina University, Greenville, NC

**Keywords:** auricular composite chondrocutaneous graft, nasal alar rim notching, nasal alar rim retraction, alar rim defect, ear composite graft

## DESCRIPTION

**Figure 1:**
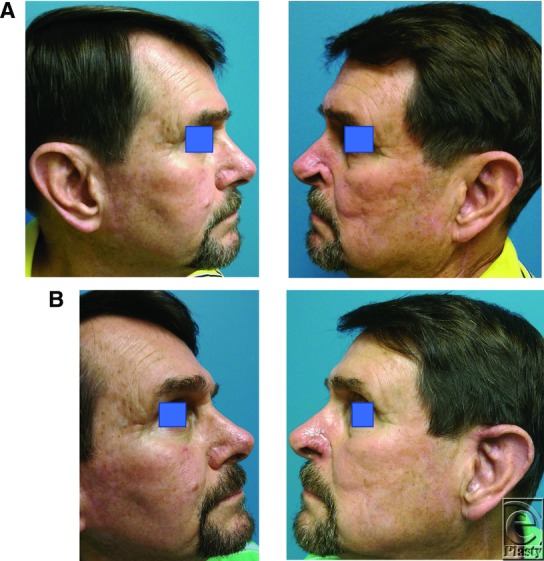
(a) Preoperative photograph demonstrating left alar notching and retraction secondary to a previous Mohs surgery performed in the alar groove/nasal sidewall. Left and right sides are compared. (b) Three-week postoperative photograph demonstrating left alar reconstruction with an ipsilateral helical root composite chondrocutaneous graft. The well-healed left ear donor site.

We present a case of a 72-year-old male patient who was referred to us with a 1.2 x 0.6-cm left alar rim notching and retraction defect (Fig 1). The alar distortion was a result of a previous basal cell carcinoma Mohs micrographic surgery in the left nasal alar groove/nasal sidewall, that is, not involving the alar rim. Mohs reconstruction led to subsequent contracture and distortion of the unoperated alar rim. We reconstructed the alar rim distortion using an auricular composite chondrocutaneous graft.

## QUESTIONS

1. Where in the ear can chondrocutaneous graft be harvested for nasal reconstruction?
2. What is the upper limit for a chondrocutaneous graft before the graft viability is compromised and what can be done to overcome composite graft failure?
3. What is the postoperative appearance sequelae of chondrocutaneous graft take?
4. What is the typical postoperative care?

## DISCUSSION

**Figure 2:**
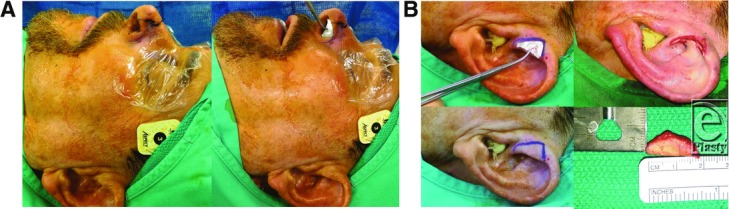
(a) Intraoperative photograph demonstrating the design of left alar defect template using a foil of a suture pack. (b) Intraoperative photograph demonstrating the harvest of a left helical root composite chondrocutaneous graft. The template is used as a guide to design a nostril margin graft. Note that the composite graft is slightly larger than the actual defect and at the time of graft inset, the skin component was cut back while leaving cartilage extension to be buried in the subcutaneous pocket.

**Figure 3:**
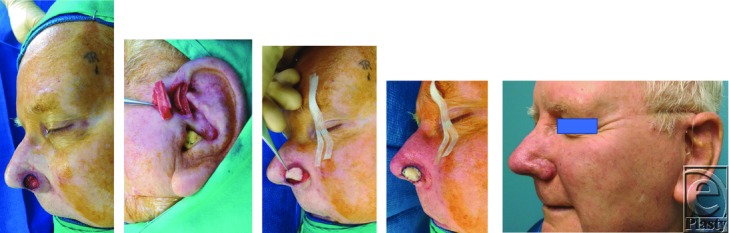
Intraoperative and 3-month postoperative photographs of a different patient demonstrating a helical root composite chondrocutaneous graft. Skouge's tongue-in-groove technique was implemented to increase the probability of graft survival and to maintain the alar rim integrity. Composite graft with cartilage struts extending beyond the cutaneous portion of the graft is fixed in small subcutaneous pockets created laterally within the soft tissues of the alar base and medially under the skin of the tip. The patient was able to wear his hearing aid postoperatively with no difficulty.

Nasal defects in the alar rim are challenging to reconstruct. Thin skin coverage, cartilage support, and thin lining are needed to replace this cosmetically prominent site. Several reconstructive choices are available for this region, but the best cosmetic result is achieved with a composite full-thickness graft from the ear. The auricle is an excellent donor source because it provides a graft with thin skin attached to a delicate segment of contoured cartilage. The cartilage provides structural support and the skin that closely resembles the adjacent nasal skin of the nostril margin.^1^ Composite auricular grafts are typically harvested from the helical root (Figs 2 and 3) but can be harvested from throughout the ear such as the helical rim, helical crus, antihelix, concha, fossa triangularis, scapha, tragus, and antitragus.^1^^,^^2^

Auricular composite chondrocutaneous grafts have high metabolic demands. Composite cartilage grafts only interface with the recipient bed around the graft's perimeter. The traditional recommendation is to limit the size of composite grafts to 1 cm or less due to limited blood flow, which otherwise will lead to tissue necrosis of the graft.^1^^,^^3^^-^5 However, considerably larger grafts may be successful if they are placed in a vascular recipient site and the graft is designed so that no portion is more than 1.0 cm from the wound edge. Skouge^6^ advocated a tongue-in-groove technique when using composite grafts. This technique involves harvesting the graft with cartilage struts extending beyond the borders of the soft tissue and inserting the border of the graft between 2 layers of tissue at the recipient site^6^ (Figs 2 and 3). This method of graft attachment has the effect of increasing graft stability and increasing surface contact between graft and recipient site for revascularization.^1^^,^^6^ Other perioperative strategy recommended by some authors to increase graft take includes allowing the defect to heal by secondary intention, followed by delayed reconstruction with composite graft. In such instances, a hinged flap of scar and epithelium is formed during the secondary healing, which is dissected to increase the surface area for attaching a composite graft. This has shown a greater success rate. Other adjunctive measures to increase the take of a composite graft include surface cooling with iced compresses, which decreases the metabolic demand of the graft; hyperbaric oxygen therapy, which promotes fibroblast replication, collagen formation, and neovascularization; and pre- and postoperative corticosteroids.^3^

**Figure 4:**
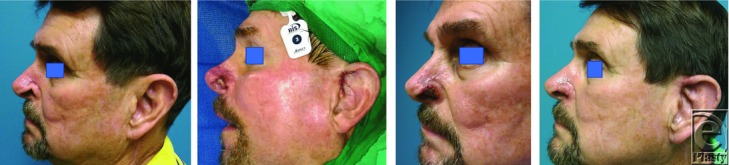
(a) Preoperative left alar rim defect. (b) Pale appearing composite graft immediately postoperatively, sutured into place. (c) Seven-day postoperative congested/cyanotic appearing composite graft. (d) Three-week postoperative. Composite graft containing skin and cartilage, harvested from the helical root, closely matches the color, contour, and thickness of the recipient side.

Composite chondrocutaneous grafts follow a predictable healing pattern. Successful grafts transition in color during the first week: white, then blue, and then progressively pink/red. Immediately following introduction to local anesthetic and harvesting from the ear, the composite graft will appear pale (Fig 4*b*) due to lack of sufficient vasculature; the graft obtains its nourishment through plasma imbibition. However, as it progresses toward inosculation around 1 to 2 days postoperatively, it will acquire a congested blue color (Fig 4*c*), denoted as deep cyanosis, and even crustiness.^7^ Over the course of 3 to 7 days, cyanosis will clear and healthy pink color will return as revascularization improves^7^^,^^8^ (Fig 4*d*).

Patient education and postoperative care are extremely important. Upon discharge, patients are given the following instructions: 10 minutes on/10 minutes off ice-saline compress application for the first 1 to 2 days; minimize all activities; avoid nose blowing; sneeze with an open mouth; and wipe gently with tissue. Bacitracin should be applied with a Q-tip regularly and gently around the incision inside and outside the nose. Teeth should only be brushed with a soft toothbrush. Avoid manipulation of the upper lip and excessive smiling for 1 to 2 weeks. Only wear clothing that fastens in the front or the back, and avoid slipover sweaters, tight T-shirts, and turtle necks. The only dietary restriction related to the alar rim composite graft reconstruction is avoiding foods that require prolonged chewing. Washing the face gently is permitted, as long as avoiding touching the nose. Finally, avoid sun exposure and a hot environment for 6 weeks postoperatively.

## References

[1] Son D, Kwak M, Yun S, Yeo H, Kim J, Han K (2012). Large auricular chondrocutaneous composite graft for nasal alar and columellar reconstruction. Arch Plast Surg.

[2] Hendi A (2006). Reconstruction of an alar rim defect. Dermatol Surg.

[3] Kim G, Jeong Y-I, Shim H-C (2014). Auricular composite chondrocutaneous grafts in the repair of nasal alar rim defects. Ann Dermatol.

[4] van der Eerden PA, Verdam FJ, Dennis SCR, Vuyk H (2009). Free cartilage grafts and healing by secondary intention: a viable reconstructive combination after excision of nonmelanoma skin cancer in the nasal alar region. Arch Facial Plast Surg.

[5] Son D, Jeong H, Choi T (2010). A new mechanism associated with composite graft success. J Plast Reconstr Aesthet Surg.

[6] Skouge JW Skin Grafting.

[7] McLaughlin CR (1954). Composite ear grafts and their blood supply. Br J Plast Surg.

[8] Brown JB, Cannon B (1946). Composite free grafts of two surfaces of skin and cartilage from the ear. Ann Surg.

